# Craniobot: A computer numerical controlled robot for cranial microsurgeries

**DOI:** 10.1038/s41598-018-37073-w

**Published:** 2019-01-31

**Authors:** Leila Ghanbari, Mathew L. Rynes, Jia Hu, Daniel S. Schulman, Gregory W. Johnson, Michael Laroque, Gabriella M. Shull, Suhasa B. Kodandaramaiah

**Affiliations:** 1 Department of Mechanical Engineering, University of Minnesota, Twin Cities, Minnesota, USA; 2 Department of Biomedical Engineering, University of Minnesota, Twin Cities, Minnesota, USA

**Keywords:** Neuroscience, Biomedical engineering

## Abstract

Over the last few decades, a plethora of tools has been developed for neuroscientists to interface with the brain. Implementing these tools requires precisely removing sections of the skull to access the brain. These delicate cranial microsurgical procedures need to be performed on the sub-millimeter thick bone without damaging the underlying tissue and therefore, require significant training. Automating some of these procedures would not only enable more precise microsurgical operations, but also facilitate widespread use of advanced neurotechnologies. Here, we introduce the “Craniobot”, a cranial microsurgery platform that combines automated skull surface profiling with a computer numerical controlled (CNC) milling machine to perform a variety of cranial microsurgical procedures on mice. The Craniobot utilizes a low-force contact sensor to profile the skull surface and uses this information to perform precise milling operations within minutes. We have used the Craniobot to perform intact skull thinning and open small to large craniotomies over the dorsal cortex.

## Introduction

The palette of tools available for systems neuroscientists to measure and manipulate the brain has exploded in the last decade. *In vivo* neuroscience experiments have evolved from using simple electrical sensing and stimulation tools, such as tungsten electrodes^1^ to using multi-channel silicon-based 3-dimensional (3D) electrodes^2,3^. Recent advances in fabrication techniques and materials have enabled the development of flexible neural interfaces^4–6^, injectable neural mesh electrodes^4^, and 3D organic electrodes^7^. In parallel, the emergence of optical tools such as optogenetic molecules^8–11^ and fluorescent activity reporters^12,13^ have enabled researchers to investigate wide regions of the brain at cellular or near-cellular resolution^14,15^.

As neurotechnologies have advanced, the corresponding cranial microsurgery procedures to deploy them have become more complex. For example, multi-shank neural probes require precisely arrayed craniotomies for insertion into regions of interest. Calcium (Ca^2+^) imaging was initially performed through chronically implanted planar glass coverslips over 3–4 mm diameter craniotomies^16^. With the emergence of wide field-of-view imaging technologies, neuroscientists have progressed to implanting coverslips across a whole hemisphere of the cortex^17,18^, and more recently, curved glass windows across the whole dorsal cortex^14^. Intact skull preparations are also commonly used for optical imaging on mice, and are particularly useful in studies where neuro-inflammatory effects need to be minimized^19–21^. These procedures require the skull to be thinned down to tens of micrometers.

Mice, the most widely used mammalian model organism, have very thin skulls, typically ranging from 100–650 μm above the dorsal cortex (Supplementary Fig. 1). Any surgical procedure involving bone removal requires great care and precision, ensuring the underlying dura and brain tissue are not damaged. Further, the quality of the procedure significantly affects the success of experiments. For instance, *in vivo* patch clamping experiments rely on pristine craniotomy preparations, for high success rate^22–25^.

Cranial microsurgery procedures are typically performed using tools adapted from dentistry such as handheld dental drills fitted with burrs. Because these procedures are performed manually, they are imprecise and require several months of training. Consequently, many of the more advanced neurotechnologies are confined to a select few lab groups. Automating some of these procedures would potentially enable more precise cranial microsurgeries and facilitate the wider use of advanced neurotechnologies.

Several attempts to automate craniotomies have been made, such as using force or impedance feedback to control the drilling depth^26–28^. Force feedback based systems are typically designed for large animal models and have not been used for automating microsurgical procedures in mice. Impedance sensing feedback^27^ achieves micrometer scale precision and has been demonstrated in mice, but its performance is affected by the vasculature in the skull and is not generalizable across many areas of the skull. Further, these methods are constrained to complete removal of bone for craniotomies and cannot be used for partial bone removal as in the case of intact skull preparations^19,20^. Other versions include using femtosecond lasers to remove skull tissue^29^, which would be impractical to implement broadly due to high cost. A generalized methodology for performing a wide range of microsurgical procedures is currently not available. If such a methodology can be derived and implemented in an inexpensive robotic system, it could be a valuable resource for systems neuroscientists.

Here, we introduce the “Craniobot”, a comprehensive microsurgery platform for mice based on a modified desktop computer numerical controlled (CNC) mill. CNC machining practices have been ubiquitously used in manufacturing and enable a diverse range of precise machining processes. The Craniobot combines automated skull surface profiling with CNC milling to perform precise microsurgical procedures such as intact skull thinning, small to large craniotomies of arbitrary shapes on the dorsal skull, and drilling pilot holes for anchoring cranial implants.

## Results

### Principles of CNC microsurgery

**Figure 1:**
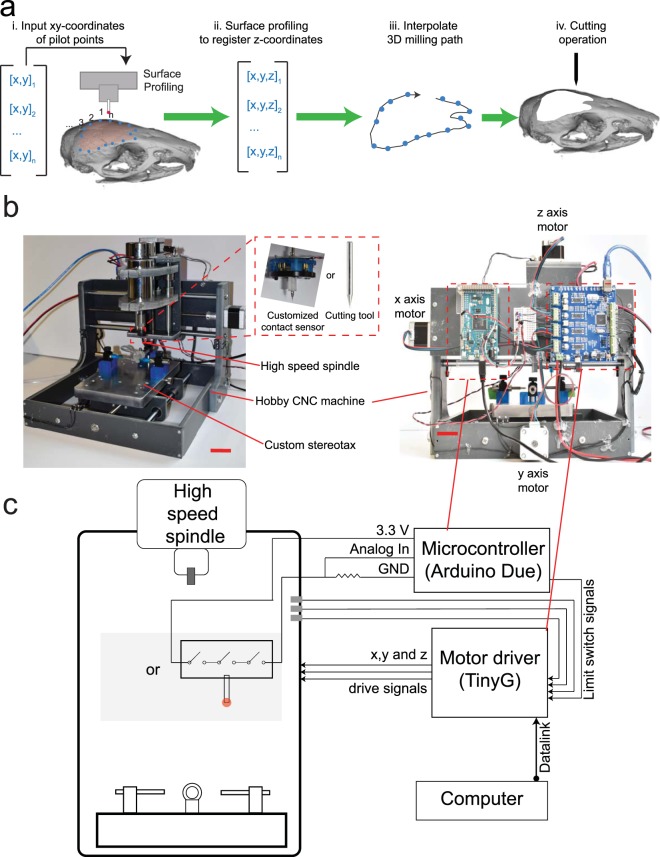
Principles of surface-profile-guided computer numerically controlled (CNC) skull machining: (**a**) (i) Given a set of xy-coordinates of the pilot points along the desired craniotomy, (ii) a device can be used to register the z-coordinates at each point. (iii) This information can be used to interpolate a 3-dimensional (3D) milling path. (iv) The 3D path can be used by a CNC machining tool to iteratively mill the skull until it is thin enough to be fractured and excised. (**b**) We built the ‘Craniobot’ to perform this algorithm for automated microsurgical procedures by adapting a CNC milling machine typically used for woodworking. A 3-axis milling base guides a high-speed spindle which can accommodate either a contact sensor for profiling the skull surface or a cutting tool for machining. It incorporates a custom-built stereotax into the mill base. Open-source motor drivers and microcontrollers are integrated to execute the Craniobot algorithm. Scale bar, 4 cm. (**c**) A schematic illustrating the electronic components in the Craniobot.

We hypothesized that if an apparatus could be built to precisely determine the coordinates of pilot points along a cutting path on the skull surface, it would be possible to interpolate a 3D cutting trajectory that can be executed precisely by a CNC mill (Fig. 1a). Iterative milling could be used to thin down the skull until it could be excised via fracturing. The method of excising the skull would be similar to the procedure commonly used in craniotomies^30^, but the CNC milling would dramatically improve the precision and control of the bone tissue removal process. The process of iterative milling would avoid damaging the underlying dura and brain as long as the final depth of milling was less than the minimum thickness of the skull at any point along the cutting path.

We established an automated surgery platform, “Craniobot”, to perform automated surface profiling along with iterative milling procedures (Fig. 1b,c). The Craniobot consists of a 3-axis CNC milling machine, typically used by woodworking hobbyists. Further, the Craniobot can be operated using open-source G-code scripts. The milling machine includes a spindle that can either be fitted with a contact sensor or a milling tool. We incorporated a custom built stereotax in the bed of the CNC mill to secure the mouse in the Craniobot (Supplementary Fig. 2). The Craniobot enabled profiling the skull surface at pilot points along a pre-defined trajectory encompassing a majority of the dorsal skull. Once the surface profiling probe contacted the skull at each pilot points, the z-coordinate was registered at that point. These registered coordinates were used to interpolate a 3D cutting path for milling the skull.

We first heuristically determined appropriate depth of milling by measuring the skull thicknesses from micro-computed tomography (micro-CT) scans of C57BL/6J mice. Across the dorsal cortex, the average thickness of the skull was 245 µm and the thickness ranged between 100–650 µm (n = 3 adult mice over 8 week old, Supplementary Fig. 1), with high thickness values mostly being measured at the midline suture. Mouse skull sutures by ~45 days of age, and most cranial expansion is complete by 6 weeks^31,32^. Thus we can anticipate these skull thickness measurements to generalize across a particular strain. We set the initial milling pass at 50 µm, or half the minimum thickness measured in the micro-CT scans. Subsequently, the milling depth was increased in steps of 10–15 µm with each milling pass, so that the craniotomy could be performed in a precisely controlled fashion until the skull was fragile enough to be excised (Supplementary Note 1). For initial testing of the concept, we built a simple motorized-manipulator guided end mill and incorporated it into a standard rodent stereotax (Supplementary Fig. 3). We found that the drilling could be reliably stopped with at least 50 µm thick skull still remaining and excise the bone to complete the craniotomy procedure (Supplementary Fig. 4, Supplementary Note 1).

### Automated profiling of skull surface

**Figure 2:**
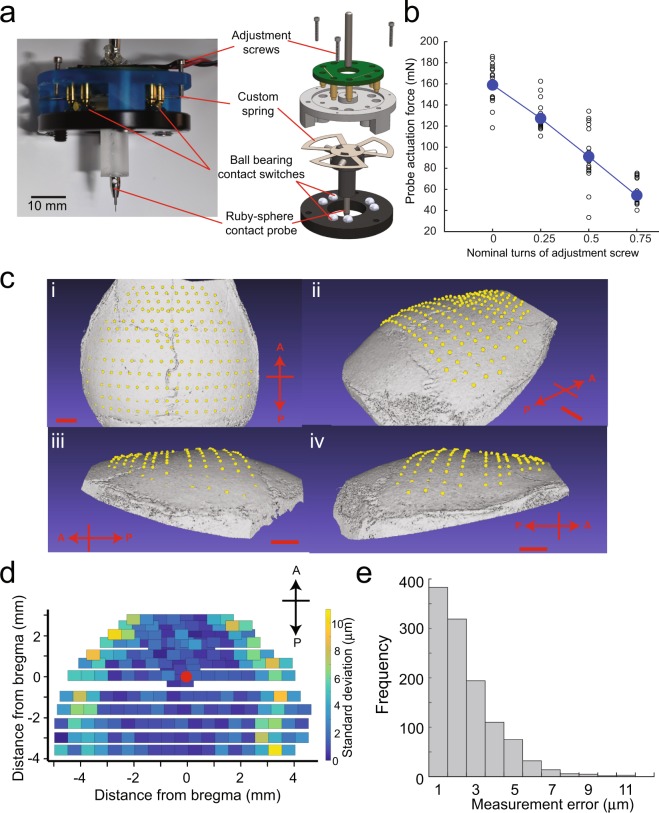
Automatic skull surface profiling: (**a**) We engineered a low-force contact sensor for profiling the surface of the skull. Incorporating a custom spring (Supplementary Note 2) enabled the contact sensor to accurately detect the skull surface without causing any visible deflection when observed through a stereomicroscope at 60X. Scale bar, 10 mm. (**b**) The relationship between the probe actuation force required to detect contact with the skull and the nominal turns of the adjustment screws. The actuation force ranged from 49 to 98 mN in all experiments. The average actuation force from 20 measurements is shown for each quarter turn of the adjustment screws as a filled circle and the individual measurements are shown as hollow circles. (**c**) The average point-cloud generated by surface profiling the dorsal skull of an 8-week old male C57BL/6J mouse with a needle tip stylus at 192 points overlaid on the micro-CT scan of the same skull (i) top view, (ii) side view from the left, (iii) side view from the right, and (iv) isometric view. Scale bar, 1 mm. (**d**) Distribution of the measurement error as a function of the distance from bregma. (**e**) The cumulative histogram of the measurement errors for n = 6 mice (n = 3 males and n = 3 females).

CNC machining relies on precise measurement of substrate surfaces, typically accomplished using a contact sensor with a digital readout. We modified a commercially available contact sensor typically used for profiling metal substrates by incorporating a custom-designed linear spring (Fig. 2a,b, details in Supplementary Note 2) to have 49–98 mN actuation force. This range of actuation force allowed us to accurately detect contact with the skull surface without any perceptible deformation, as visualized using a stereomicroscope (Supplementary Video 1).

We tested the performance of the low force contact sensor by profiling a 2-inch gauge block and a 0.850-inch gauge pin and compared its performance to an industrial touch probe (Supplementary Fig. 5). The average measurement error when using the low force contact sensor on the 2″ gauge block was 0.7 µm, while the measurement error with the industrial touch probe was 1.0 µm. Similarly, on the 0.850″ gauge pin, which had a curved surface, we found the measurement errors to be 1.1 µm and 0.8 µm for the low force contact sensor and the industrial touch probe respectively. We found no significant difference between their performance on either the gauge block or the gauge pin (p = 0.33 on block, p = 0.15 on pin, t-test).

Next, to determine how well the contact sensor could profile the surface of the skull, we profiled the skulls of 6 mice (n = 3 male, n = 3 female C57BL/6J mice) and evaluated the distribution of measurement errors. We defined an xy point map consisting of 192 points spanning the skull spread across the parietal and frontal bones, covering the whole dorsal cortex. The contact sensor was equipped with either a needle tip stylus or a ruby sphere tip stylus. We then used the contact sensor to profile the skull surface at each point. We performed five complete scans on each mouse and calculated the average z-coordinate measured at each of the 192 points. These coordinates were used to construct an average xyz point cloud. The average point cloud from a representative mouse superimposed on the 3D surface generated via a micro-CT scan of its skull made a conforming fit (Fig. 2c). This was a qualitative indication that the contact sensor could accurately measure the topology of the dorsal skull surface.

We then quantified the precision of the contact sensor. For each point, we calculated the measurement error and examined its variation as a function of the distance from bregma (Fig. 2d). The maximum measurement error across all measurements was 11 μm (n = 6 mice, 3 males, and 3 females). Over 97% of the measurements had an error of less than 6 μm (Fig. 2e). The average measurement error was 1.7 ± 1.4 μm for female mice (n = 3 mice), and 2.2 ± 1.8 μm for male mice (n = 3 mice). There was no significant difference in the mean error between male and female mice (p = 0.62, t-test). Qualitatively, higher errors were observed at lateral extremes on the skull surface. As a result, we examined the effect of the instantaneous slope of the skull on the measurement error, particularly with the ruby sphere stylus, as it is more susceptible to forces acting at an angle to the normal axis. There was a slight correlation between the measurement error and the instantaneous slope on the skull surface (r = 0.18). If the Craniobot needed to be used in the future on surfaces with steeper curvatures than in the experiments in this study, it would be possible to alleviate this issue by incorporating a tilt axis such that the contact sensor approached the skull normal to the surface. Overall, there was no significant difference between the measurement error of the contact sensor when equipped with the ruby sphere stylus tip versus when equipped with the needle stylus tip (p = 0.13, t-test). Thus, the contact sensor could be used to precisely map most of the skull surface spanning the dorsal cortex.

### Surface profile guided machining

**Figure 3:**
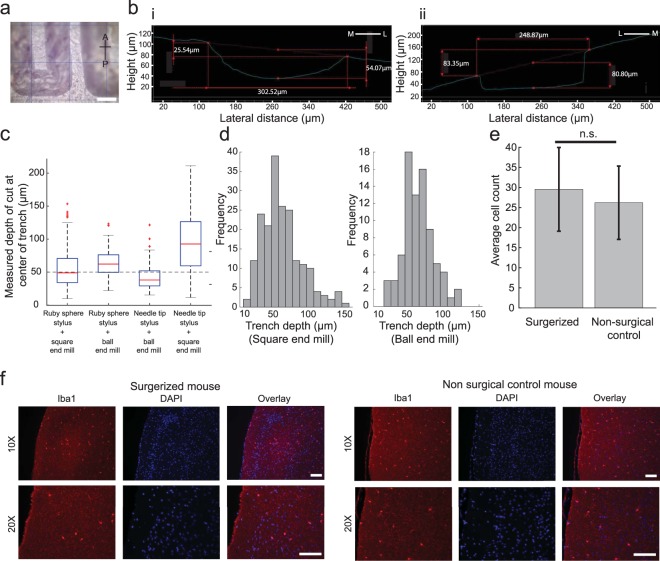
Characterizing performance of surface-profile-guided CNC machining of the skull: (**a**) Photomicrograph of the 50 μm deep test milling path on the right hemisphere ~4 mm posterior to bregma and ~1.5 mm lateral to midline, drilled using the 300 μm ball end mill. Grid size, 500 µm; scale bar, 250 µm (**b**) Cross-sectional profile of the trenches drilled with the (i) 300 μm ball end mill and (ii) the 200 μm square end mill constructed from the photomicrographs. (**c**) Boxplot of the measured trench depth for each of the four possible combinations of surface profiling and cutting tools. The dashed line represents the target depth of cut, in contrast with the median value for each combination shown in red. (**d**) Histograms of trench depths for milling with the square end mill and the ball end mill after surface profiling with the ruby sphere stylus. (**e**) Average microglia cell counts in tissue near the cortical surface of area 891 × 662 µm as imaged at 10X magnification in mice that underwent automated surface milling and non-surgical control mice. (**f**) Representative images of stained microglia via Iba1 staining and DAPI shown in both mice that underwent automated surface milling and non-surgical control mice. Images were taken at 10X magnification (top row) and 20X magnification (bottom row). Scale bars, 100 µm.

Once we established that we had a robust and accurate means of profiling the surface of the skull, we examined how well this information could be used to guide the CNC mill to perform microsurgical procedures. To do so, we had to account for the dimensions of the stylus used for surface profiling and the dimensions of the cutting tool. We designed a milling path that guided a CNC tool to mill 50 µm deep trenches along the anterior-posterior axis at various distances spaced 500 µm from the midline suture (Fig. 3a). Surface profiles were generated by equipping the contact sensor with either a needle tip stylus or a ruby-tip stylus. A 200 µm square end mill and a 300 µm ball end mill resembling commonly used dental burs, were used to mill 50 µm deep trenches using the surface profile information (Fig. 3b, Table 1). The average cutting depth across all combinations of surface profiling styluses and cutting tools was 59.3 ± 16.2 μm. Three of the four combinations resulted in trench depths close to the desired value of 50 μm, with the ruby sphere stylus and the 200 μm square end mill combination performing closest to target. The combination of the needle tip stylus and the 300 μm ball end mill resulted in an average trench depth of 44.2 ± 5 μm (n = 3 mice, 78 measured points). We found that the average trench depth was 96.4 ± 19 μm for the combination of the needle tip stylus and 200 μm square end mill (n = 1 mouse, 27 measured points). Using the ruby sphere for surface profiling in combination with the 300 μm ball end mill resulted in an average trench depth of 64.0 ± 5 μm (n = 2 mice, 86 measured points). The combination of ruby sphere tip with the 200 μm square end mill resulted in average trench depth of 57.3 ± 6 μm (n = 3 mice, 77 measured points) (Fig. 3c). The trench depth measurements were taken at randomly chosen locations, independent of the locations of the pilot points. In our preliminary investigation (Supplementary Note 1, the average difference between the drilling depth and minimum thickness of the skull was 56.2 µm in C57BL/6 mice, and 147.2 µm in Thy1-GCaMP6f mice (Supplementary Fig. 4). Thus, the precision of the Craniobot was sufficient to safely perform a wide range of microsurgical procedures. Based on average trench depths measured, the final experiments for performing craniotomies and skull thinning used the ruby-sphere stylus for profiling and either the square end mill or the ball end mill for milling. The histogram of trench depths for these two conditions are shown in Fig. 3d.

**Table 1:** Summary of trench milling depth measurements.

Surface profiler	Cutting tool	Average trench depth (μm)	# of mice	# of measured points
Needle tip stylus	300 μm ball end mill	44.2 ± 5	3	78
Needle tip stylus	200 μm square end mill	96.4 ± 19	1	27
Ruby sphere tip	300 μm ball end mill	64.0 ± 5	2	86
Ruby sphere tip	200 μm square end mill	57.3 ± 6	3	77

We next assessed if automated skull machining caused any acute injury to the brain. To do this, the Craniobot was used to perform surface profile guided milling of a 3 mm diameter circular craniotomy to a depth at which the remaining skull island was loose enough to be fractured and removed. At this point, the milling was stopped and the mice were kept anesthetized for one hour to allow for any microglia aggregation at the cortical surface that occurs in response to acute injury. The mice were then transcardially perfused to fix the brains. We performed immunohistochemical staining for ionized calcium binding adaptor molecule 1 (Iba1, Fig. 3e,f) found in microglia. Tissue sections of size 891 × 662 µm at the cortical surface directly underneath the automated milling procedure had a mean cell count 29.5 ± 10.43 cells in mice that underwent surgery (n = 6 mice) and was 26.17 ± 9.1 cells in non-surgical control mice (n = 6 mice). There was no statistically significant increase in microglia cell count (p = 0.08, t-test). No heat induced blood-brain barrier opening below the drill path was investigated^33^. Thus, we can conclude that the Craniobot’s milling procedure does not result in acute injury to the brain.

Further, we analyzed whether the instantaneous slope of the skull surface in the medial-lateral direction affected the depth of trench drilling in each of the four conditions. Only the combination of ruby sphere probe and the 300 μm ball end mill displayed a significant relationship between the trench depth and the slope in the medial-lateral direction (p = 0.008, linear regression). In the other three conditions, there was no statistically significant evidence that the slope of the skull affected the depth of cut. Based upon these results, we concluded that the Craniobot is not only capable of precisely mapping the skull surface, but it is also able to perform precise machining operations using this information across a wide swathe of the dorsal skull.

### CNC microsurgical procedures performed using the Craniobot

**Figure 4:**
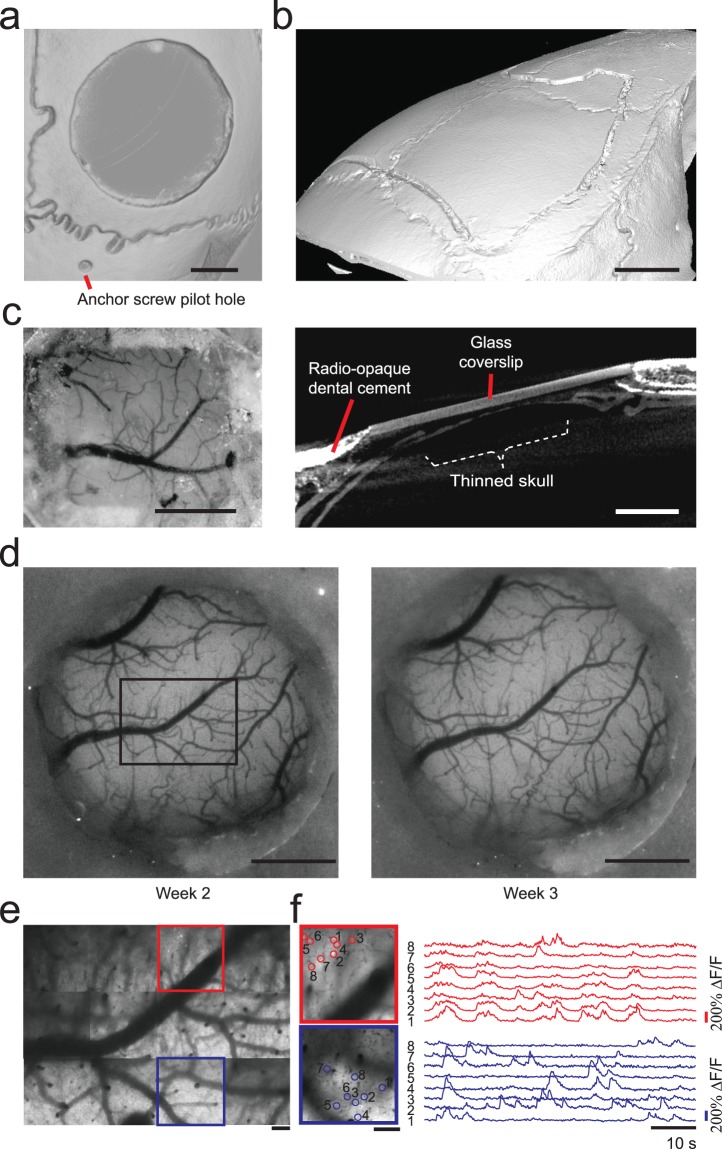
Demonstration of the Craniobot surgical capabilities: (**a**) A micro-CT scan of an adult male C57BL/6J mouse skull after performing an automated circular craniotomy, drilling an anchor screw pilot hole, and fully excising the skull island. Scale bar, 1 mm. (**b**) A micro-CT scan of an adult male C57BL/6J mouse skull after milling a 50 µm deep trench for a craniotomy over the majority of the dorsal cortex. Scale bar, 2 mm. (**c**) Left: A photograph taken from an adult male C57BL/6J mouse skull after a thinning operation using the Craniobot and reinforcing it with clear dental cement and a glass coverslip. Surface milling was performed to a depth of 70 μm in a 2 × 2 mm square. Right: A micro-CT scan of the same mouse. A cross-sectional view of the thinned portion of the intact skull implanted with a glass coverslip. Scale bar, 1 mm. (**d**) Photographs taken from a female Thy1-GCaMP6f mouse with a chronically implanted circular glass coverslip. Scale bars, 1 mm. (**e**) Composite image of multiple maximum intensity z-projection images constructed from z-stacks acquired using a 2P microscope from adjacent 365 × 365 µm tiles in the black rectangular area highlighted in **d**. Scale bars, 100 µm. (**f**) Left: Average intensity image constructed from 60 s long time series acquired in the tiles highlighted in red and blue squares in **e**. Scale bar,100 µm. Right: Time series traces of Ca^2+^ activities of individual neurons highlighted by open circles in the average intensity images.

We performed a range of cranial microsurgery procedures using the Craniobot. A demonstration of a 3.3 mm wide craniotomy followed by skull excision to expose the primary somatosensory cortical area is illustrated in Fig. 4a. Profiling the skull surface at 40 points took 4 minutes and 40 seconds, while each surface milling iteration took ~50 seconds. We started the initial milling at a depth of 50 µm, followed by an inspection of the bone to examine if it was fragile enough to be excised. If not, we performed additional milling operations, with each subsequent milling run having the depth incremented at 10–15 µm. The final drilling depth was 81.87 ± 24.35 µm (n = 6 C57BL/6J mice and n = 2 Thy1-GCaMP6f). Thus, the cutting operation was typically completed within 2–3 milling iterations and took no longer than 4 minutes. Milling down to a depth of 50 µm resulted in anchor holes for skull screw implantation. We could successfully excise the bone in all cases without causing damage to the underlying tissue as indicated by lack of bleeding or blebbing at the brain surface. Thus, this is a relatively quick and high-throughput procedure as compared to manual craniotomies.

The Craniobot was programmed to perform craniotomies over the skull above the whole dorsal cortex (Fig. 4b, Supplementary Fig. 4). This could enable the chronic implantation of large cranial windows for wide-field optical imaging^14^. The milling path was designed to avoid major sinuses that are present along midline 2–3 mm anterior to bregma. Skull surface profiling was performed at 97 points that took 11 minutes with each milling pass taking 60 s. The milling operation did not through the entire skull at any point along the milling path as shown in Fig. 4b.

High-resolution imaging can also be done through the intact skull by thinning and polishing it down to 20–30 µm followed by reinforcement with transparent dental acrylic cement^19,20^. The efficacy of this technique is however highly dependent on the surgeon as any unevenness in the thinned skull surface may lead to mottled images. This is another process that could benefit from automation. We programmed the Craniobot to collect skull surface coordinates from 169 pilot points in a 2 × 2 mm section to guide surface milling. Each point, took ~7 seconds to profile, thus, the surface profiling took ~20 minutes and the surface milling operation took ~4 minutes. Figure 4c shows a photograph of a mouse skull thinned to the depth of 70 μm. Reinforcing the skull with clear dental cement, followed by capping with a glass coverslip allows chronic optical access to the cortex, with clearly discernible micrometer diameter blood vessels visible through the thinned skull.

We also verified that craniotomies performed by the Craniobot could be used to implant circular glass coverslips for chronic optical access to the cortex (Fig. 4d). The cranial windows remained clear without any clouding or bone regrowth for multiple weeks, allowing high-resolution optical imaging using a two-photon (2P) microscope on Thy-GCaMP6f mice (Fig. 4e). We created a composite image by compiling maximum intensity projections of z-stacks acquired down to depth of 200 µm from the pial surface. Anatomical features in the composite image, such as blood vessels, matched those in the macroscopic images taken earlier (Fig. 4d). Individual neurons and Ca^2+^ activity could be readily discriminated in time-series images acquired at depths of 50–200 µm (Fig. 4f). Thus, high-resolution 2P imaging could be performed over a large area of the implant.

## Discussion

We demonstrated a generalized, robotic microsurgery platform, the Craniobot, which combines automated surface profiling with CNC milling to perform a variety of cranial microsurgical procedures to provide access to the brain in a fast and systematic fashion. Procedures can be performed with micrometer-scale precision independent of the 3D morphology of the skull without damaging the underlying tissue. The Craniobot can perform craniotomies for implantation of chronic cranial windows, skull thinning for optical imaging through the intact skull, and drilling pilot holes for bone anchor screw implantation. For simple craniotomies, it provides significant increases in speed. Compared to other automation efforts^27,34^, our setup has ~2 times increase in speed for performing circular craniotomies. Thus, the Craniobot is a high-throughput microsurgery platform which lowers entry barriers and reduces both training time and cost of performing intricate microsurgical procedures, thereby democratizing the use of advanced neurotechnologies.

It can also be programmed to perform challenging craniotomies across the whole dorsal cortex. While the surface profiling for skull thinning took longer, several parameters could be modified to optimize the process, such as by increasing the speed of the contact sensor when approaching the skull, reducing the number of pilot points, decreasing the retraction distance after profiling a point or using a milling tool with larger contact areas.

In this study, we have demonstrated the utility of the Craniobot for performing procedures on the skull above the whole dorsal cortex. The same methodology could be extended beyond the dorsal cortex for automated microsurgery above the cerebellum. In the future, we could explore extending the Craniobot to perform bone tissue removal procedures on organs that have much more complex topologies such as the spinal cord. Given the precision of the Craniobot, it could also be extended to smaller animals with delicate skulls such as songbirds or juvenile mice, which are key for studying development.

The Craniobot is fully open-source and uses a high-level machine language, G-code, to automate microsurgical procedures. Given that G-code programming is ubiquitous in manufacturing and precision machining, the programming environment is highly developed. Thus, the Craniobot has access to massive libraries of automation scripts and programs. This infrastructure also enables the user to modify parameters such as the end-mill speed. In the future, more complex microsurgical procedures could be explored by harnessing these resources which are currently not available to existing motorized stereotaxes that require proprietary software and drivers. Further, a critical difference between the Craniobot and existing motorized stereotaxes is that the components that keep the mouse immobilized in the Craniobot are attached to the bed of the mill, and translates in the anterior–posterior direction, while the cutting tool translates in the medial-lateral and z-direction.

The Craniobot hardware is inexpensive since the entire setup is built using off-the-shelf components for a fraction of the cost of commercially available stereotaxes. Modifications of the hardware, for instance, integrating a tilt axis for machining highly curved surfaces could be easily accomplished. In the future, additional functionalities such as bone flap removal, fluidics for irrigating tissue during surgery, control of virus injections, insertion and implantation of devices could all be integrated with relatively simple modifications to the hardware.

Currently, optical imaging is predominantly confined to mice, because of highly developed genetic strategies for expressing optical reporters and optogenetic molecules^35–39^. With the advent of new genetic tools such as CRISPR, it will be possible soon to generate genetically modified rats and non-human primates (NHPs), allowing the study of complex cognitive behaviors that are not possible in mice^40,41^. Extending cranial microsurgical procedures optimized in mice to imaging experiments for these species will be very useful, and automation tools such as the Craniobot can streamline that process. In NHPs, access to structural imaging data such as CT and magnetic resonance imaging (MRI) scans, can be used for depth co-registration along with the surface profiling for a safe milling procedure. Additionally, force-feedback chucks could be used to automatically disengage the cutting tool in the rare instances when the cutting tool penetrates the bone completely. These advances could further ensure the Craniobot’s safety and reliability, potentially translating to clinical use.

Robots such as the Craniobot reduce experimental variability and enhance throughput. This could bolster emerging industrial scale neuroscience initiatives such as the Blue Brain project^42^, Connectomics^43^, and Allen Brain Atlases^44^. By automating cranial microsurgeries, the Craniobot could streamline these neuroscience pipelines and enable the systematic generation of vast datasets.

## Methods

### The Craniobot Construction and Design

#### The mill framework

A 3-axis hobby machining mill (Lukcase LC8110) was used as the base machine for the Craniobot. The mill is widely sold by a variety of manufacturers online and is generically called a 2020B CNC mill, which refers to the size of the xy bed of the machine (20 × 20 cm). The frame of the mill was modified to include a home-built custom stereotax in place of the stock mill bed (Supplementary Fig. 2). 3D positioning was accomplished by three NEMA 17 stepper motors and M8 stainless steel lead-screw assemblies.

#### Custom stereotax

The custom stereotax was constructed by first machining a base out of a 0.75″ thick aluminum block using a water jet cutter with added abrasives. Holes were water jet cut, drilled, and then tapped into the aluminum base to fit the stereotax components. The entire assembly was mounted on the mill base. The base was finished with a wire wheel brush to smoothen out the surface. The stereotax parts were made via a combination of 3D printed parts, off-the-shelf components, and machined parts (Supplementary Fig. 2).

#### Control electronics

We replaced the factory electronics included in the hobby mill with an open-source stepper motor controller (TinyG v8, Synthetos). It combines a microcontroller (Atmel ATxmega192) and four stepper motor drivers (TI DRV8811) onto a single printed circuit board and connects to the computer via universal serial bus (USB) interface. An additional microcontroller (Arduino Due, Atmel ATSAM3X8E) was used to connect the contact sensor to the stepper motor controller.

#### Software

Communication to and from the machine was accomplished over USB and formatted using standardized CNC G-code language packaged inside JavaScript Object Notation (JSON) text strings. The entire robot is operated through a software suite we created in Python 3.6 (Python Software Foundation) which generates, sends, and receives commands to communicate with the Craniobot. First, the software generates the xy-coordinates of a craniotomy based upon user inputs (i.e. a 3 mm diameter circular craniotomy offset 1.5 mm from bregma, or a whole-dorsal skull craniotomy with a custom shape) and formats these in G-Code-based JSON strings. The Craniobot then uses the customized contact sensor to scan for the z coordinate at each point.

#### Contact sensor

A custom contact sensor modified from an industrial digitizing surface probe (Tormach, SPU-40) is monitored via analog input from a microcontroller (Arduino Due). The contact sensor electronics consist of three normally closed switches connected in series. Each switch consists of two spherical stainless-steel contacts electrically bridged by a brass cylindrical arm. The three arms are pressed into the probe tip assembly, and this assembly nominally rests on the three sets of spherical ball contacts creating a normally closed switch circuit. When the probe tip contacts the mouse skull, one of the arms lifts off the spherical contacts and open the circuit. The internal customized spring presses the tip assembly onto the contacts and improves contact resistance. Additionally, we 3D printed a new housing that allowed us to adjust actuation force given by a customized flat spring with #0–80 screws. The resulting design is illustrated in Fig. 2a.

### Surgical procedure

All animal experiments were conducted in accordance with approved University of Minnesota Institutional Animal Care and Use Committee protocol. Characterization and demonstration experiments were conducted in acute anesthetized conditions. Mice (C57BL/6J and Thy1-GCaMP6f mice, 8–14 weeks old, Jackson Laboratories) were administered buprenorphine (Par Pharmaceutical, Chestnut Ridge, NY) at 1 mg/kg body weight, and meloxicam (Dechra Veterinary Products, Overland Park, KS) 1–2 mg/kg body weight at the time of the experiment. Mice were anesthetized with 1–5% isoflurane (Piramal Critical Care Inc., Bethlehem, PA), shaved of scalp fur, and placed on the custom stereotax. Standard aseptic techniques were used to sterilize the scalp area before the incision. The skin and fascia were then carefully removed around the targeted cutting path. All procedures were performed with aseptic techniques. For characterization experiments, mice were euthanized and perfused immediately upon the completion of the procedure. This was followed by a variety of experiments that were used to characterize the performance of the contact sensor and the ability of the CNC mill to use the surface profiling information in microsurgical procedures and demonstration experiments.

For microsurgical procedures involving chronic implantations, slow-releasing buprenorphine (Buprenorphine SR LAB, Zoopharm, Windsor, CO) at 1 mg/kg body weight was administered along with meloxicam two hours before the experiment. 3.3 mm diameter circular craniotomy was performed using the Craniobot. 4 mm diameter glass coverslip (Deckgläser, Marienfeld-Superior Inc.) was placed over the craniotomy and glued using cyanoacrylate glue (Vetbond, 3 M, Saint Paul, MN). Dental cement (S380, C&B Metabond, Parkell Inc.) was then applied to secure the coverslip to the skull surface. We performed chronic implantations of 4 mm glass coverslips in 3 mice using this procedure. After the implantation, the mice were recovered on a heating pad and monitored until they could move autonomously. Meloxicam was administered to mice for 3 days after the experiment along with general monitoring for signs of pain.

### The Craniobot Operation

The Craniobot functions via automating two major processes: CNC profiling the skull surface and using that information to perform CNC milling. Both procedures are described in the following sections.

#### Surface profiling procedure

A flowchart illustrating this procedure is shown in Supplementary Fig. 6. To profile the skull surface, the needle tip (II M2 030 03 015, ITP Styli LLC, St Louis, MO) or ruby sphere (TH M2 003 03 010, ITP Styli LLC, St Louis, MO) stylus were sterilized with 70% ethanol, washed with 0.9% sterile saline, and attached to the end of the custom contact sensor. The contact sensor was then mounted on the Craniobot’s spindle and was positioned 2–5 mm above the bregma by sending jog commands to the Python software suite. The contact sensor was used to measure the pilot point coordinates in the milling path, which were typically spaced a maximum distance of 0.5 mm. The Craniobot moved the contact sensor down at a speed of 5 mm/min while constantly sensing the switch signal attached to the sensor. Once a contact was detected, the bregma coordinates were registered as the origin of a Cartesian coordinate system. Once instructed to begin surface profiling along the cutting path, the Craniobot systematically profiled the z coordinate at each pilot point.

#### Milling procedure

A flowchart illustrating the milling process is shown in Supplementary Fig. 6. First, the contact probe was replaced by a cutting tool. We used a 200 μm (13908, Harvey Tool Inc. diameter square end mill, and a 300 μm ball end mill (24512, Harvey Tool Inc.) as cutting tools. The tip of the end mill was then carefully brought in contact with the skull surface at bregma while observing under a stereomicroscope. This process is depicted in Supplementary Fig. 7. Then, the experimenter registered bregma as the origin of a new coordinate system used for the milling procedure. The Craniobot then guided the cutting tool through the 3D milling path generated from surface profiling. Saline was used to remove tissue debris during the milling process. At the end of the milling procedure, the cutting tool was raised up 2 mm above the skull surface and stopped. At this point, the experimenter could inspect the engraved/excised skull and decide to stop the milling operation or start another iteration by increasing the milling depth. To perform craniotomies using the Craniobot, the milling depth was increased incrementally until the skull along the cutting path was thin enough to be fractured at any one segment along the mill path. Lifting off the skull island using forceps at this point results in the fracture propagating along the rest of the milled path releasing the skull island. By performing surgeries this way, the milling depth never exceeded the minimum thickness of the skull along the milling path.

### Perfusion

Following acute surgical procedures, mice were either euthanized or transcardially perfused to preserve the tissues for future analysis. Perfusions were performed by first ensuring the animal was deeply anesthetized with 5% isoflurane at 0.6 L/min pure Oxygen. Phosphate-buffered saline (PBS) (CAT# P5493-1L, Sigma Aldrich) was used to flush the circulatory system at a volume of 1 mL/g body weight, and then 4% paraformaldehyde (PFA, CAT# P6148-500G, Sigma Aldrich) was used to fix the tissues. Immediately after the procedure, the skull of the animal was collected and preserved in a solution of 4% PFA.

### Micro-CT scanning

We used micro-CT to analyze and reconstruct the 3D images of the skull after the microsurgical procedures characterization. Perfused mouse skulls were cast in acrylic (Dentsply Orthodontic Resin and Caulk, York, PA, USA) on a Teflon pedestal, and imaged using a Micro-CT machine (XT H 225, Nikon Metrology Inc., Brighton, MI, USA). X-rays were generated with parameters of 105 kV, 85 µA. Mouse skulls were scanned using 720 projections at a half degree pitch while recording 4 frames per projection. Samples were then returned to a solution of 4% PFA after scanning. The micro-CT software reconstructed the x-ray images into a volume graphics (.vgi) file. VG Studio MAX 3.0 (Volume Graphics GmbH, Heidelberg Germany) was used to de-noise unwanted signal caused with a bandpass filter and visualize the 3D structure of the scanned skulls. The 3D structure of the scanned skulls was then transformed into a cloud of 3D coordinates and then exported as a stereolithography (.stl) file.

### Characterizing custom low force contact sensor performance

To test the accuracy of the contact sensor’s measurements, a 2-inch gauge plate and 0.85-inch gauge pin machined with a very low tolerance were surface profiled in a 1.5 × 1.5 mm square array at 16 points (4 × 4 points). Each point was measured five times using the low force contact sensor and an industrial touch probe. The five measured points were then used to calculate the measurement error at each location. The same origin was used for each experiment by fixing the gauge pin and gauge block to the bed of the machine.

### Characterizing CNC machining precision

To investigate how the Craniobot’s surface profiling affects the depth of trench milling at different locations on the skull surface, we designed a stereotypical test path. The maximum spacing between pilot points was 0.5 mm, and the trenches were separated by 0.5 mm. Surface profiling was carried out using either the needle tip or the ruby sphere tip stylus, and the milling operation was performed using either a 200 μm square end mill or a 300 μm ball end mill (four conditions). After milling, the mice were immediately perfused, and the skull surface was gently cleaned using sterile saline to remove bone debris from the trenches, after which the skulls were preserved in 4% PFA.

A digital microscope (Keyence VHX-5000E, at 1500X, 2.1 μm resolution) was used to acquire z-stacks and reconstruct a 3D image of the skull surface in multiple areas. We also used the 3D image to calculate the skull slope in the medial-lateral (dz/dx). Measurements were performed every 500 μm, except when residuals such as bone fragments were present.

### Ca^2+^ imaging and data analysis

The mouse was allowed to recover from surgery for 7 days before imaging experiments.

A 2P microscope (Leica SP5II) with a 25X (0.95 Numerical Aperture) objective was used for cellular resolution imaging experiments *in vivo*. The laser was tuned to 940 nm excitation wavelength. The mouse was head-fixed under the 2P microscope in a custom designed disk treadmill. Locations in the FOV were targeted at 12 adjacent 365 × 365 μm tiles (with an overlap of 25 µm) in the awake mouse (Fig. 4e). Z-stacks (512 × 512 pixels) were acquired every 10 µm from the pial surface down to the depth of 200 µm. Time series of Ca^2+^ activities were acquired at 20 Hz (256 × 256 pixels) for 1 minute at a plane at depth of 50–150 μm from the pial surface.

The collected data was imported to Fiji^45^. Maximum projection images were obtained from the z-stacks and average intensity images from the time series. Average intensity images were used to locate the individual neurons in that plane. A custom MATLAB code was used to calculate and normalize the fluorescent intensity changes in a subset of the neurons. Baseline fluorescence intensity was calculated based on average over the first 4 seconds (80 frames) of each time series. Finally, the normalized data was filtered using a Butterworth filter (2-pole Butterworth low-pass filter: 0.3 Hz)^46^.

### Histology

The Craniobot milling procedure was performed on a subset of mice (n = 6) iteratively until the surgeon could remove the skull flap and complete the craniotomy. Mice were left on the stereotax for 1 hour after the milling was complete to allow possible migration of microglia to the site of the milling in case of acute injury. The mice were transcardially perfused according to the procedure described above. Coronal sections of the brains of 10 µm thickness were acquired at the location of the milling procedure. Fixed brain sliced were quenched and rehydrated using standard procedures. For antigen retrieval, slides were incubated in 6.0 pH buffer (Reveal Decloaking reagent, Biocare Medical, Concord, CA) in a steamer for 30 min at 95–98 °C, followed by a 20 min cool down period. Endogenous peroxidase activity was quenched by slide immersion in 3% hydrogen peroxide solution (Peroxidazed, Biocare) for 10 min followed by TBST rinse. A blocking solution Rodent Block M, (Biocare Medical, Concord, CA) was placed on sections for one hour at room temperature. Blocking solution was removed and slides were incubated in primary antibody diluted in 10% blocking solution/90% TBST. Rabbit polyclonal anti-Iba-1(WAKO, Richmond,VA;1:500), were incubated over night at 4 degrees. The following day slides were TBST rinsed and AlexFluor555 (Life Technologies, Eugene, OR,1:500) was applied and incubated in the dark for 2 hours at room temperature. All slides then proceeded with TBST rinse times two, followed by a distilled water rinse. Slides were then cover slipped using ProLong Gold anti-fade reagent with DAPI. Mounted slices were imaged using an inverted epi-flourescence microscope (Eclipse Ti2, Nikon). In each mouse, average microglia cell counts were obtained in tissue near the cortical surface of area 891 × 662 µm as imaged at 10X magnification. This experiment was repeated in mice that did not undergo any surgical manipulation as a control (n = 6).

### Software Availability Statement

Current versions of software are available for download at: www.github.com/bsbrl/craniobot.

## Supplementary information

Supplementary Video 1

Supplementary Figures and Notes

Supplementary Dataset 1

## Data Availability

Source data used to generate all plots have been have all been provided with the manuscript in ‘Supplementary Dataset.xlsx’. All other data supporting the findings of this study are available from the corresponding author on request.
