# Supplementary material for: Craniobot: A computer numerical controlled robot for cranial microsurgeries
Source: Sci Rep. 2019 Jan 31;9:1023. doi: 10.1038/s41598-018-37073-w (PMC6355931; doi:10.1038/s41598-018-37073-w)
Supplement: Supplementary file 2 — Supplementary Figures and Notes [file 41598_2018_37073_MOESM2_ESM.pdf]

SUPPLEMENTARY INFORMATION

Craniobot: A computer numerical controlled robot for cranial microsurgeries

Leila Ghanbari[1]*, Mathew Rynes[2]*, Jia Hu[2], Daniel Sousa Schulman[1], Gregory Johnson[1], Michael Laroque[1], Gabriella Shull[2], Suhasa B. Kodandaramaiah[1,2]

[1]Department of Mechanical Engineering, University of Minnesota, Twin Cities

[2]Department of Biomedical Engineering, University of Minnesota, Twin Cities

Equal contribution

Supplementary Video 1: Automated profiling of a C57BL/6 mouse skull surface

SUPPLEMENTARY FIGURES AND NOTES

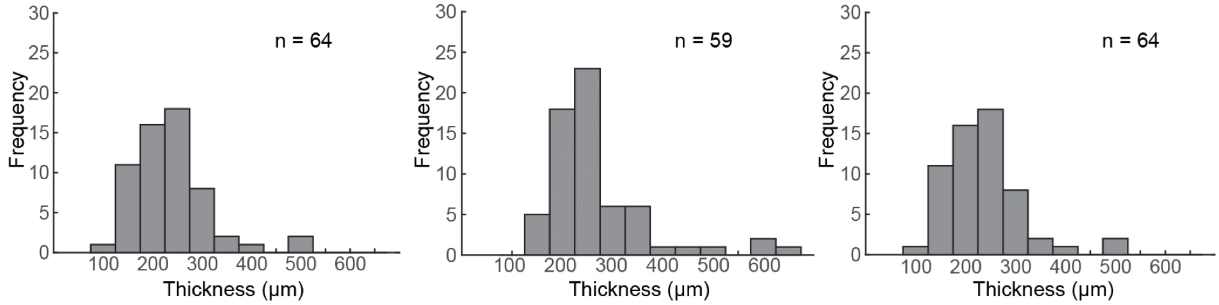

Supplementary Figure 1. Histograms of skull thicknesses measured from micro-CT scans of three mice. 64, 59, and 64 measurements were taken across the skull covering most of the dorsal cortex. The maximum skull thickness was 655 µm, and the minimum was 113 µm.

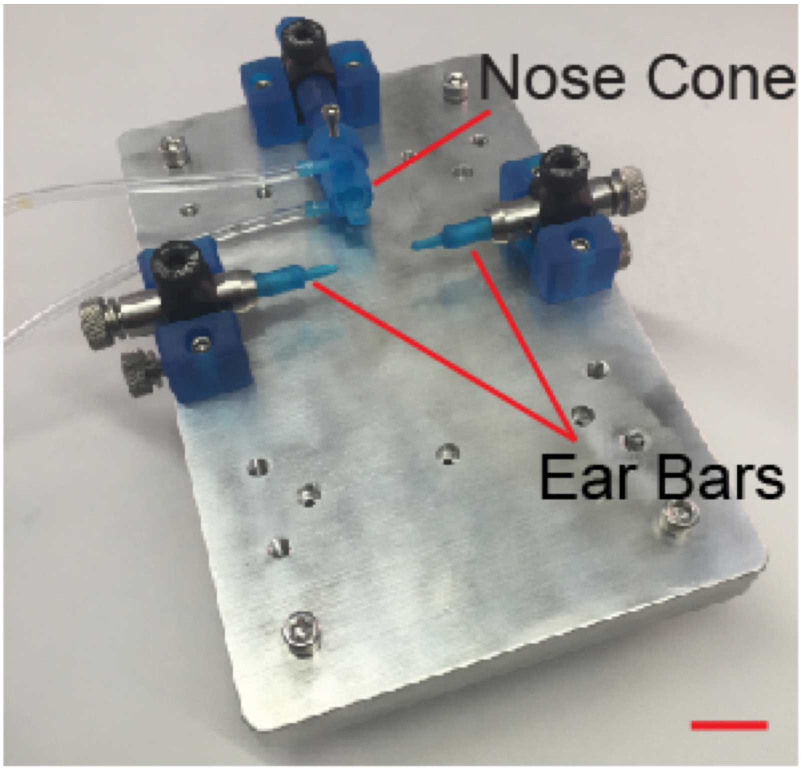

Supplementary Figure 2. Photograph of the custom-built stereotax integrated into the Craniobot's mill base. Scale bar, 2 cm.

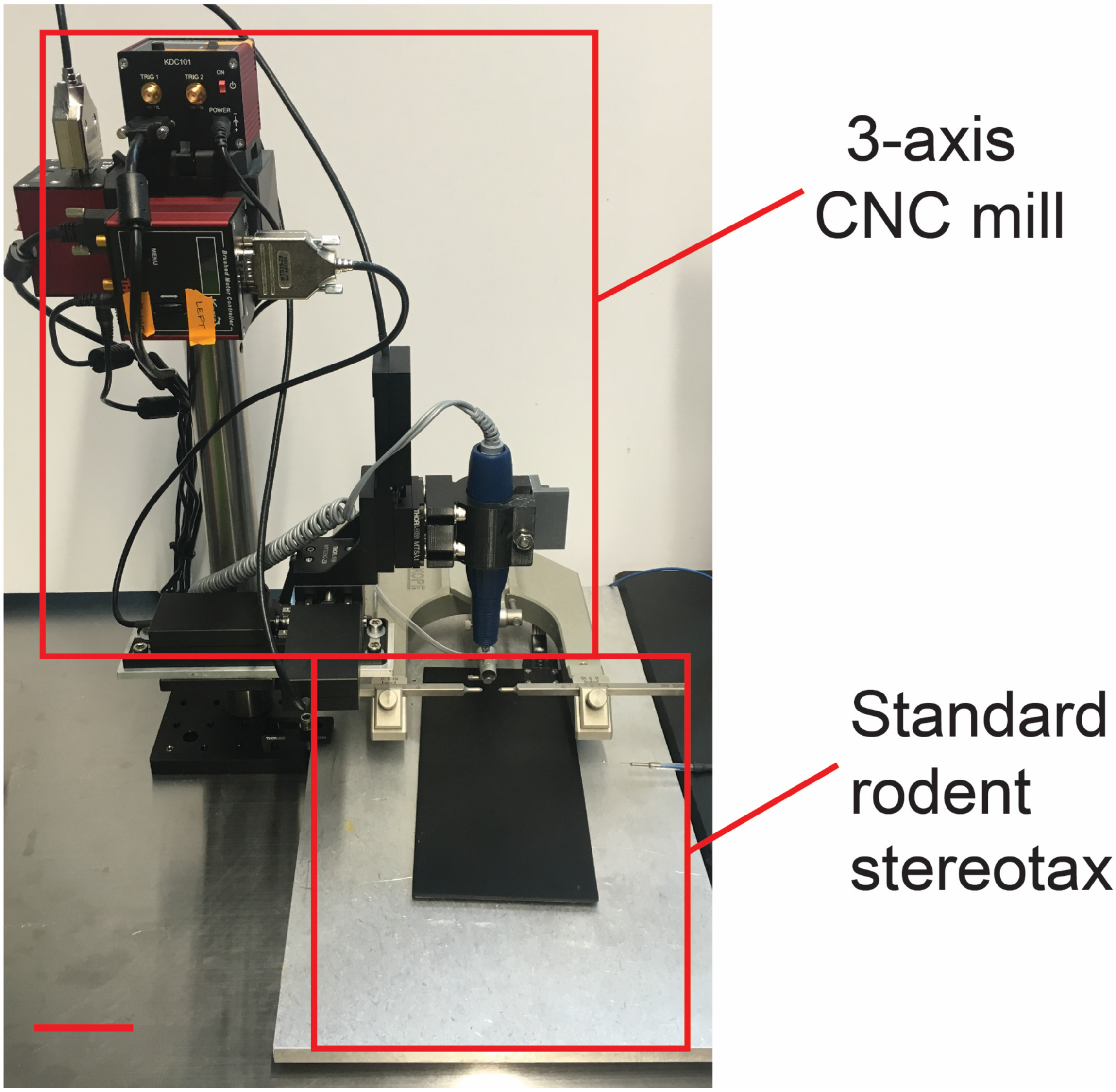

Supplementary Figure 3. Prototype craniotomy robot in a standard rodent stereotax. An end mill mounted on a motorized 3-axis programmable manipulator is attached to the stereotax. Scale bar, 5 cm.

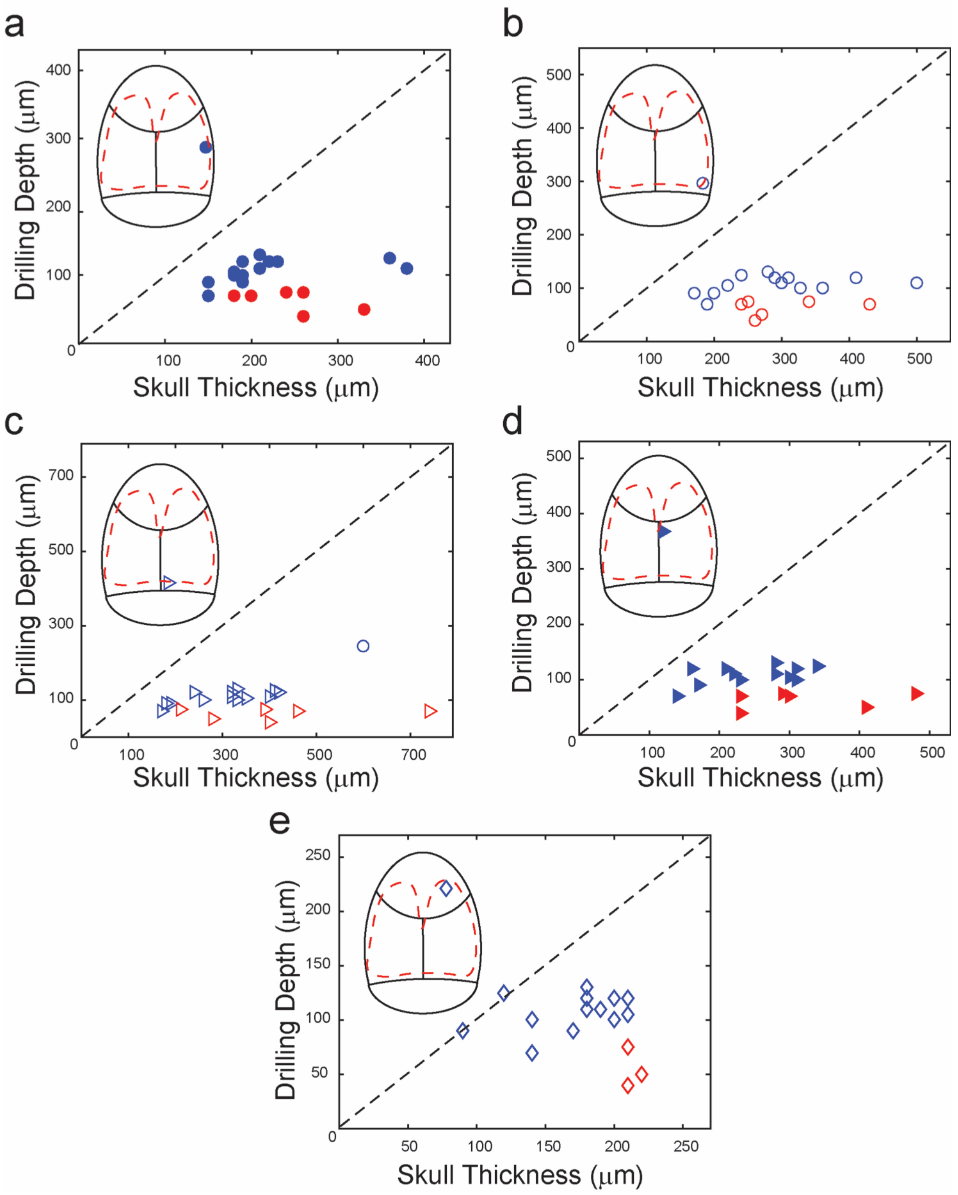

Supplementary Figure 4. Drilling depth vs. skull thickness using the prototype craniotomy robot: The thickness of the skulls at five locations indicated by the markers in the top left insets were measured after excision (**Supplementary Note 1**, n=19 mice). The drilling depth versus skull thickness is plotted with the top left insets indicating the location of the skull thickness measurements. Measurements were taken at **(a)** ~4.5 mm lateral from bregma, **(b)** ~4 mm lateral from lambda, **(c)** at lambda, **(d)** at bregma, and **(e)** ~2 mm lateral and ~2.5 mm anterior to bregma. Blue data points were collected in wild-type C57BL/6J mice of ages ranging between 7-13 weeks. Red data points were collected in Thy1-GCaMP6f mice of ages ranging between 10-14 weeks. Red dashed path indicates the boundary of the craniotomy that was performed to excise the skull prior to measurements.

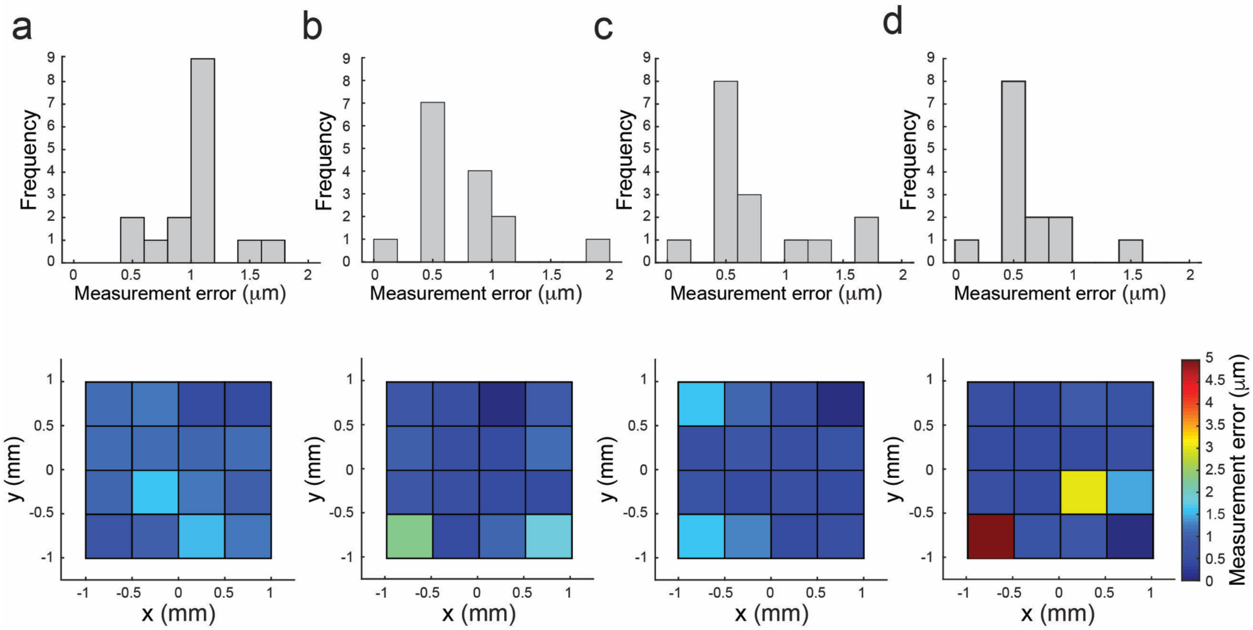

Supplementary Figure 5. Comparison of low force contact sensor performance with standard industrial touch probe. The low force contact sensor was used to measure the surface of a standard 2-inch gauge block and the round face of a 0.850-inch gauge pin in a 1.5 mm x 1.5 mm square area at 16 equidistant points. Each point was measured 5 times, and then the measurement error was calculated from the measurements. **(a)** ***Top***: histogram of error value for the experiments performed on the gauge pin with the low force contact sensor, ***bottom***: spatial distribution of measurement error in the 16 point grid. **(b)** ***Top***: histogram of error value for the experiments performed on the gauge pin with standard industrial touch probe, ***bottom***: spatial distribution of these measurement errors in the 16 point grid. **(c)** ***Top***: histogram of error value for the experiments performed on the gauge block with the low force contact sensor, ***bottom***: spatial distribution of measurement error in the 16 point grid. **(d)** ***Top***: histogram of error value for the experiments performed on the gauge block with standard industrial touch probe, ***bottom***: spatial distribution of these measurement errors in the 16 point grid.

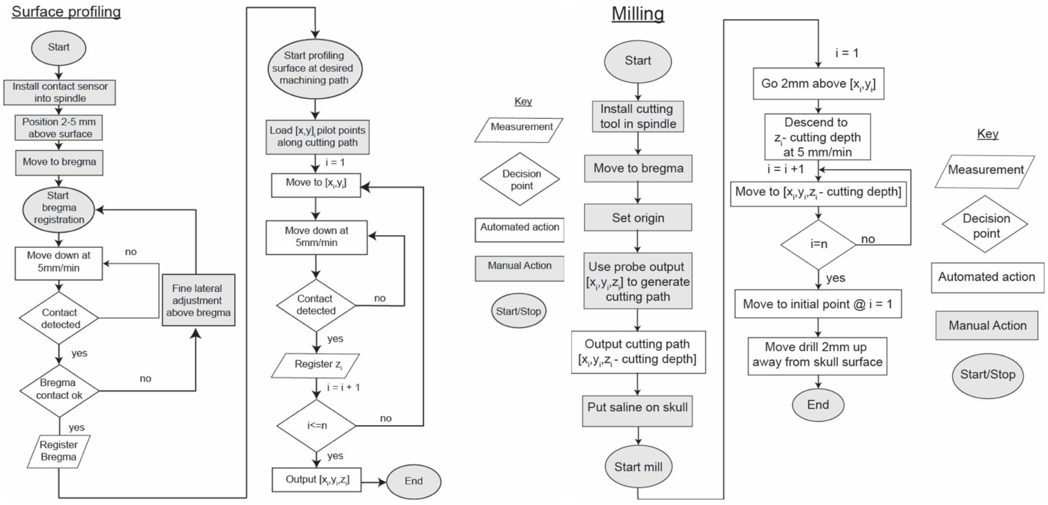

Supplementary Figure 6. Craniobot algorithm: *Left*: A detailed flowchart showing all of the steps for automated surface profiling. After the profiler is installed in the spindle, the experimenter positions it over bregma. Upon running a Python script, the Craniobot moves the contact sensor down until it meets the skull surface at bregma. If needed, fine lateral adjustments can be made to ensure proper localization to bregma. Then the bregma coordinates are registered. The x-y coordinates of the pilot points are input to the program by the experimenter, and the Craniobot uses them to guide the contact sensor to measure the z coordinate at each point. The information is then output to a Python software suite to generate a milling path. ***Right:*** After the surface profiling, the experimenter places the desired cutting tool into the spindle. The cutting tool must be then guided carefully to the surface of the skull at bregma. Then, the Craniobot uses the data output from the surface profiling process to generate a 3D linearly-interpolated milling path and guide the end mill to perform the cutting procedure.

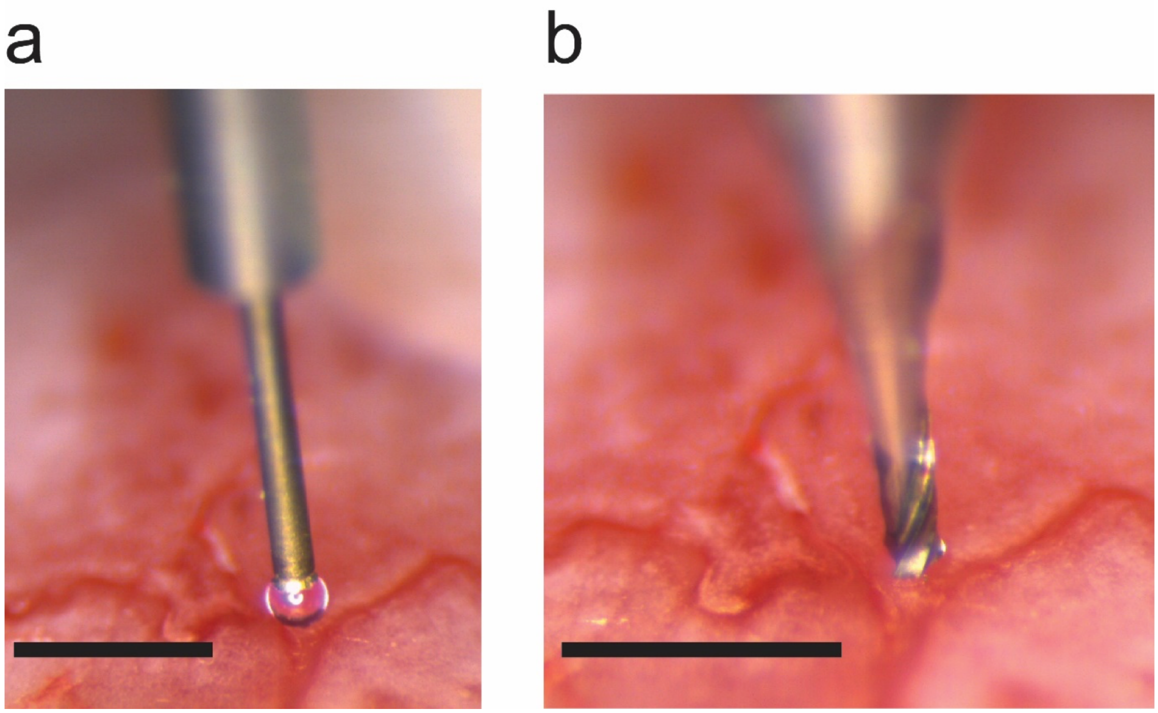

Supplementary Figure 7. Photographs depicting the protocol for homing the cutting tool after surface profiling. **(a)** A photograph of the ruby sphere tip stylus after bregma registration in contact with a mouse skull. **(b)** A photograph showing the 200 μm square end mill in contact with the mouse skull at bregma. This is performed after slight adjustments to the position of the cutting tool due to the offset of the tip on the contact sensor. Scale bars, 1 mm.

Supplementary Note 1

Preliminary testing

For initial testing of the concept, we built a simple motorized-manipulator guided end mill and incorporated it into a standard rodent stereotax (**Supplementary Fig. 3**). A handheld mill (Rampower, Ram Products Inc.) was mounted on the 3-axis stage using a custom mount. A 200 µm diameter end mill (13908, Harvey Tool Inc.) was used as the cutting tool.

A custom computer program was written in LabVIEW (National Instruments Inc.) to control this CNC robot. The first part of the code executes the skull surface profiling. Given an x-y profile of the desired craniotomy, the program guides the end mill to each pilot point. The experimenter lowers the tip of the end mill to the surface of the skull at each point and registers the z-coordinates. After all the points are registered, the program generates an interpolated 3D cutting path. The second part of the code executes an iterative milling procedure. First, the end mill machines the skull to a depth of 50 µm along the defined milling path. After this, the experimenter checks if the milled skull can be fractured along the milling path and excised. If not, this procedure is repeated by increasing the milling depth by 10 µm in each pass until the bone can be successfully removed.

We performed this CNC milling procedure in 13 wild-type C57BL/6J mice ages 7 to 13 weeks, and 6 transgenic Thy1-GCaMP6f mice ages 10 to 14 weeks. Following the craniotomy, we measured the thickness of the excised skull segment (**Supplementary Fig. 4)**. Skull thicknesses across the dorsal surface from micro-computed tomography (micro-CT) scans of C57BL/6J mice showed that the average thickness of the skull is 245 µm and the thickness ranges between 100 - 700 µm (n = 3 mice, **Supplementary Fig. 4**). The depth of milling was started at 50 µm, half the minimum thickness that we measured, and increased in steps of 10 - 15 µm with each milling pass until the skull was fragile enough to be excised. Following the craniotomy, we measured the thickness of the excised skull segments (**Supplementary Fig. 4)**. The final drilling depth was on average 56.1 ± 30.4 µm less than the minimum thickness of the excised skull segment in C57BL/6J mice (n = 13). In similar experiments conducted on Thy1-GCaMP6f mice[31], the final drilling depth was 146.6 ± 25.2 µm less than the minimum skull thickness (n = 6). This confirmed that we could use the iterative milling procedure to successfully perform craniotomies on the skull above the whole dorsal cortex without damaging the underlying tissue.

Supplementary Note 2

Design of the low force contact sensor

Commercially available contact sensors, such as Tormach SPU-40, have ~570 gram-force (5589.7905 mN) actuation force, which is too high for profiling as that force deforms the skull. We therefore modified the contact sensor by removing the stock compression spring and replacing it with a custom waterjet-cut stainless-steel spring (300 series stainless steel).

The contact sensor electronics consist of three normally closed switches connected in series. Each switch consists of two spherical stainless-steel contacts electrically bridged by a brass cylindrical contact switch. The three arms are pressed into the probe tip assembly, and this assembly nominally rests on the three sets of spherical ball contacts creating a normally closed switch circuit. When the probe tip contacts the mouse skull, one of the arms lifts off the spherical contacts and opens the circuit. When the custom spring presses the tip assembly onto the contacts, contact resistance is improved. In our Craniobot, we needed to design the spring and the pre-load on the spring such that:

(i) The contact sensor actuated only when it was in contact with the skull surface

(ii) The actuation force was not high enough to deflect the skull upon contact.

The spring consists of six radial arms connected by an arc. We modeled the spring as six parallel cantilever beams by assuming that the torsional effects in the radial arms or bending effects on the arc component are negligible. This assumption was made since the deflection of the radial arms was the most significant contribution to the spring deflection.

Based on these assumptions, the spring deflection in each cantilever arm is given by:

$$y = \frac{PL^3}{3EI}$$

where P is the force on the end of the cantilever arm, L is the length of the cantilever, E is the Young's modulus of the material, and I is the moment of inertia of the cross-section about its neutral axis.

Given that there are 6 radial arms and F is the total actuation force exerted by the skull on the contact sensor, then the spring deflection as a function of the total actuation force is given by:

$$y = \frac{FL^3}{18EI}$$

The adjustment screws (**Fig. 2a**) pre-load the springs and adjust the actuation force required to dislodge brass contact switches. The required actuation force as a function of initial spring deflection is given by:

$$\frac{F}{y} = \frac{18EI}{L^3}$$

We used #0-80 screws, which have a pitch of 3150 turns/m. Based on the dimensions of the spring used, we estimated that the actuation force would increase by ~196.133 mN/turn of the adjustment screws. We validated this by commanding the Craniobot to probe a single point on a weighing scale for multiple replicates while adjusting the screws by a quarter turn for each

condition (**Fig. 2b**). From these experiments, we found that the actual actuation force varied by ~142.196425 mN/turn. We initially tested the contact sensor on a mouse skull securely fixed on a stereotax, and found that a total force of 49.03-98.06 mN was appropriate for skull surface profiling based on visual inspection during the profiling.

The low actuation force caused the contact resistance inside the probe to be high enough (1-5 kΩ) that the probe could no longer be used in a simple passive switch circuit. We instead placed the probe in series with a 1 kΩ resistor, creating a voltage divider that was monitored using an analog input on a microcontroller. An analog voltage threshold on the microcontroller determined if the probe had been actuated and sent this information to the motor controller.

Using the contact sensor in this manner, the skull profiling operation can be fully automated by sending the motor controller an x-y coordinate, requesting the machine to probe downward at that position until the probe switch state changes, and reading back the z coordinate at which the state change happens. By repeating this process over a complete set of x-y coordinates, the skull surface contour could quickly be mapped in three dimensions for microsurgical applications.
